# Parahisian Atrial Tachycardia: Cryoablation from the Aortic Cusp

**DOI:** 10.1016/s0972-6292(16)30716-1

**Published:** 2014-01-01

**Authors:** Dursun Aras, Serkan Cay, Serkan Topaloglu, Goksel Cagirci, Ozcan Ozeke

**Affiliations:** Department of Cardiology, Division of Arrhythmia and Electrophysiology, Yuksek Ihtisas Heart-Education and Research Hospital, Ankara, Turkey

**Keywords:** Parahisian Atrial Tachycardia, Cryoablation from the Aortic Cusp

A 29-year-old female patient with Ebstein anomaly was referred to our division for drug-resistant symptomatic wide-complex tachycardia. Her preprocedural electrocardiograms showed a sinus rhythm without delta wave and a wide-complex short RP tachycardia with a rate of 150 bpm during palpitation. During conventional electrophysiologic study earliest local activation times during the tachycardia were obtained from the right parahisian region (Figure 1). A few second of initial radiofrequency ablation attempts using standard 4-mm tip radiofrequency ablation (RFA) catheter to this region resulted in cessation of the tachycardia and reversible ventriculo-atrial block. Then applying cryothermal energy using 4-mm tip cryoablation (CA) catheter (The Freezor® Cardiac CryoAblation Catheter, Minneapolis, USA) at -30 0C, cryomapping, to the initial site resulted in cessation of the tachycardia and reversible ventriculo-atrial block. The RFA catheter was advanced to the non-coronary cusp of the aortic valve which has close proximity to parahisian region [1,2]. During application of radiofrequency energy the tachycardia stopped but significant prolongation in ventriculo-atrial conduction was detected. Lastly, cryothermal energy was applied in the non-coronary cusp. Cryomapping stopped the tachycardia without any ventriculo-atrial or atrio-ventricular block (Figure 2). Therefore, CA at -70 0C was performed with 2 freeze-thaw cycles (Figure 3a and b). No tachycardia was induced with control programmed and burst pacing maneuvers and the patient was asymptomatic at 6-month follow-up.

To the best of our knowledge, this is the first case whose parahisian atrial tachycardia was ablated from the aortic cusp using cryothermal energy. Cryoablation from the aortic cusps should be considered to ablate the arrhythmia focus when RFA threatens the normal conduction system or vital anatomical structures [3].

## Figures and Tables

**Figure 1:**
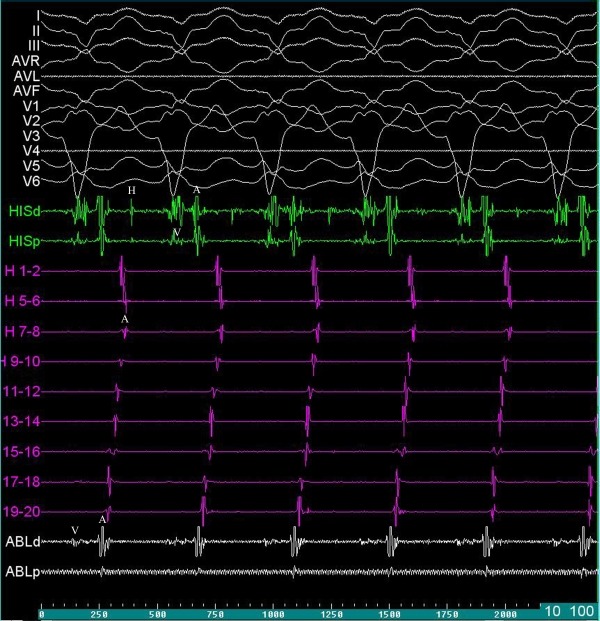
Twelve-lead surface electrocardiogram and intra-cardiac recordings showing the short RP tachycardia. A quadripolar diagnostic catheter was placed in the HIS region (HISd and HISp) showing prolonged H-V interval, short V-A interval and the earliest atrial activation; a duo-decapolar (Halo; H) catheter in the right atrium (H1-2 to H19-20) from anteroseptal region to inferior crista terminalis showing the earliest atrial activation in the anteroseptal region (H19-20) neighboring to parahisian region; and a quadripolar CA catheter in non-coronary cusp (ABLd and ABLp) showing small ventricular recordings and the earliest atrial activation compared to 'A' recording in HIS catheter. A, atrial activation; ABLd, ablation distal; ABLp, ablation proximal; CA, cryoablation; H, HIS recording; HISd, HIS distal; HISp, HIS proximal; V, ventricular activation.

**Figure 2:**
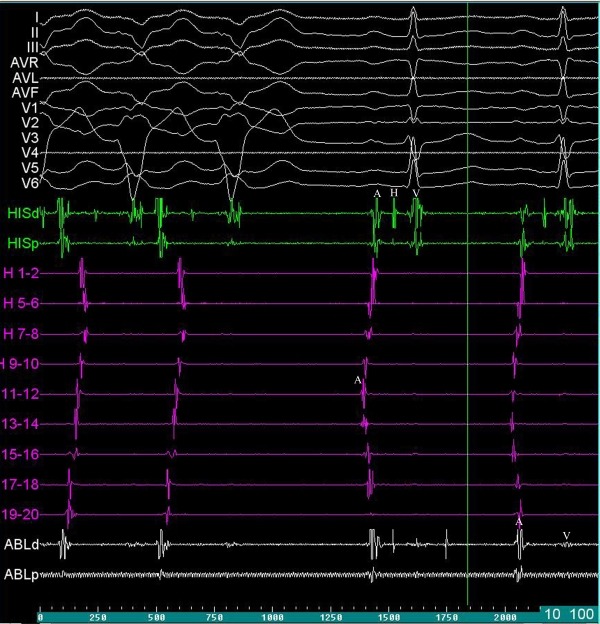
Twelve-lead surface electrocardiogram and intra-cardiac recordings during ablation of the tachycardia focus. After cessation of the tachycardia normal A-H-V intervals were seen in HIS recording (HISd); the earliest atrial activation was seen in sino-atrial node region in H11-12 bipole of Halo (H) catheter; and similar to HIS catheter late atrial activation compared to H11-12 was seen in the CA catheter (ABLd). A, atrial activation; ABLd, ablation distal; ABLp, ablation proximal; CA, cryoablation; H, HIS recording; HISd, HIS distal; HISp, HIS proximal; V, ventricular activation.

**Figure 3:**
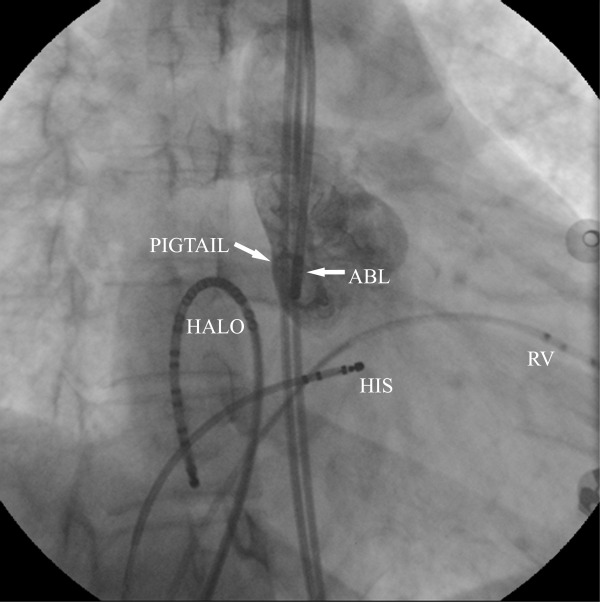

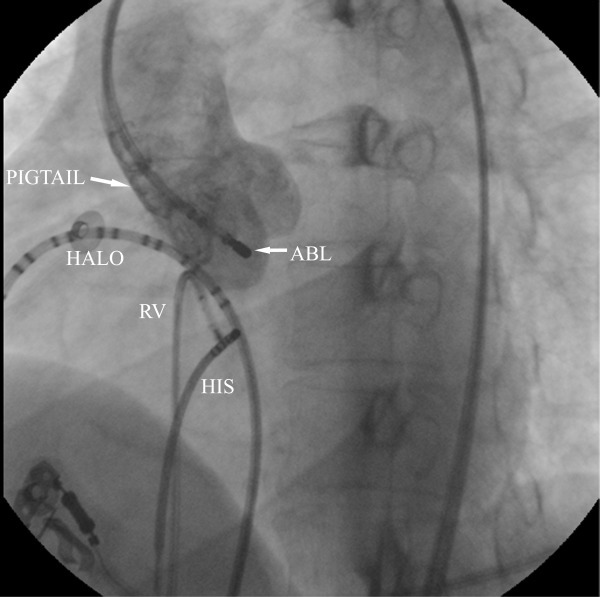
Cryoablation procedure from non-coronary cusp. Aortic root angiography was performed to localize the catheter in right anterior oblique (a) and left anterior oblique (b) projections. ABL, ablation catheter; HALO, Halo catheter; HIS, HIS catheter; PIGTAIL, pigtail catheter; RV, right ventricular catheter.
